# Successful Non-surgical Management of Cesarean Scar Pregnancy

**DOI:** 10.7759/cureus.45763

**Published:** 2023-09-22

**Authors:** Arushi Joshi, Sheila K Pillai, Usha Vishwanath

**Affiliations:** 1 Medical School, Sri Ramachandra Institute of Higher Education and Research, Chennai, IND; 2 Department of Obstetrics and Gynaecology, Sri Ramachandra Institute of Higher Education and Research, Chennai, IND

**Keywords:** cesarean scar ectopic, cesarean section, potassium chloride, transvaginal ultrasound, methotrexate

## Abstract

Ectopic pregnancy in the scar of a previous cesarean section contributes to significant maternal morbidity in the first trimester due to a significantly higher risk of uterine rupture if left undetected. The routine scans done in the first trimester serve as an important screening tool in the detection of such an ectopic pregnancy. Early detection can aid in making a paradigm shift from a surgical to a more conservative approach for the management of such pregnancies. Here, we report a case of a cesarean scar pregnancy diagnosed in the sixth week of gestation which was managed non-surgically with methotrexate and intracardiac potassium chloride injection.

## Introduction

Ectopic pregnancy or extrauterine pregnancy is the implantation of the fertilized embryo outside the uterus. Uncommon implantation sites of ectopic pregnancy include the cervix, interstitial segment of the fallopian tube, scar from a prior cesarean delivery, uterine myometrium, ovary, and peritoneal cavity [1]. Ectopic pregnancy in the scar of a previous cesarean section poses a high degree of morbidity due to the risk of vaginal or intra-abdominal bleeding, uterine rupture, and shock, which requires immediate life-saving intervention [2]. We present the case of a scar ectopic pregnancy diagnosed at six weeks and four days that was managed non-surgically.

## Case presentation

**Figure 1:**
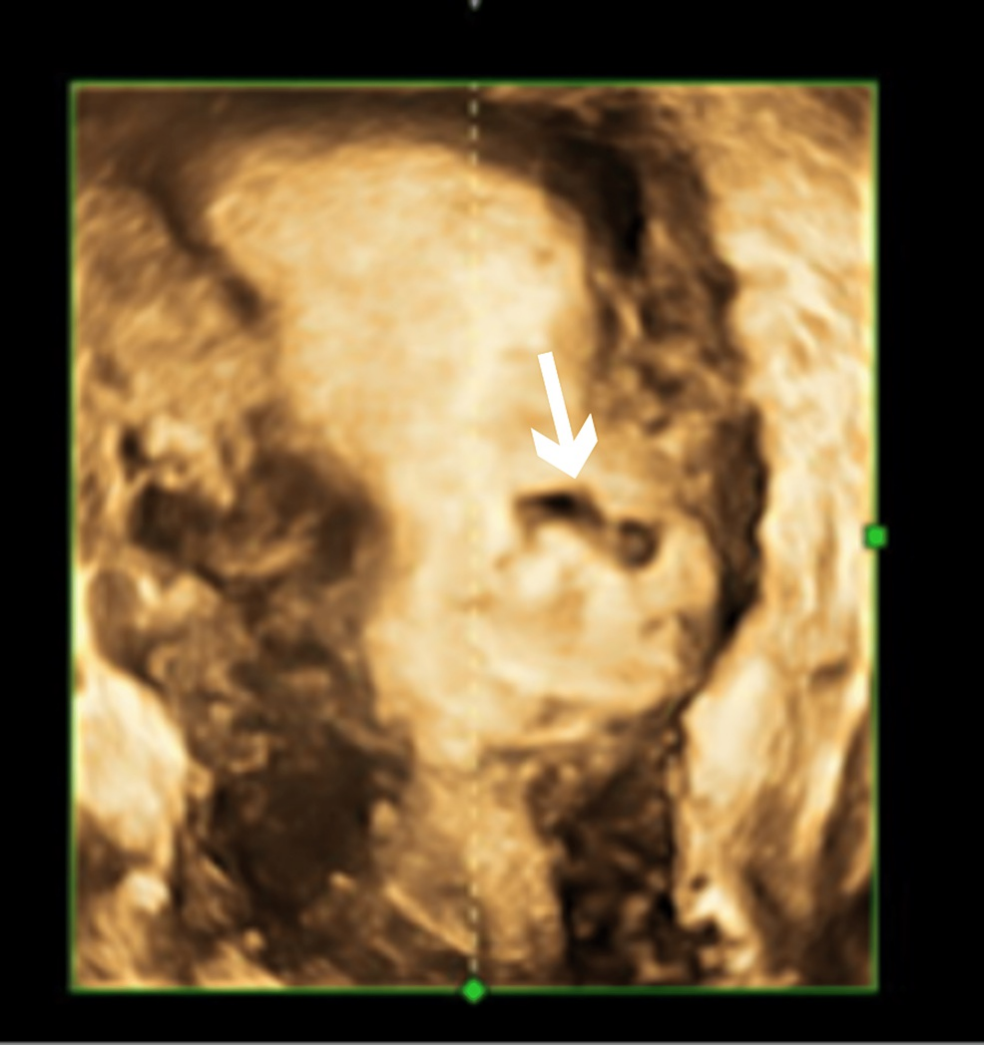
Three-dimensional ultrasound of cesarean scar pregnancy. The arrow shows the current pregnancy developing in the previous scar.

A 26-year-old G2P1L1 female presented to the Obstetrics and Gynecology outpatient department at our institution with complaints of spotting per vaginum. She had undergone a lower-segment cesarean section (LSCS) in the previous pregnancy due to fetal distress. Her spontaneous conception was confirmed by urine pregnancy at 40 days of amenorrhea. The serum beta human chorionic gonadotropin (HCG) was 11,299 IU/L. A transvaginal ultrasound (TVS) scan was done at another facility at seven weeks and one day and showed a gestational sac with a yolk sac in the scar of the previous LSCS. The patient was then admitted to our institution for in-patient management at this point. The previous pregnancy was otherwise uneventful. General and systemic examinations were found to be normal on examination of the patient, and the patient was hemodynamically stable. Her complete blood count and basic metabolic panel were normal. A three-dimensional ultrasound scan showed a cesarean scar pregnancy (Figure 1).

**Figure 2:**
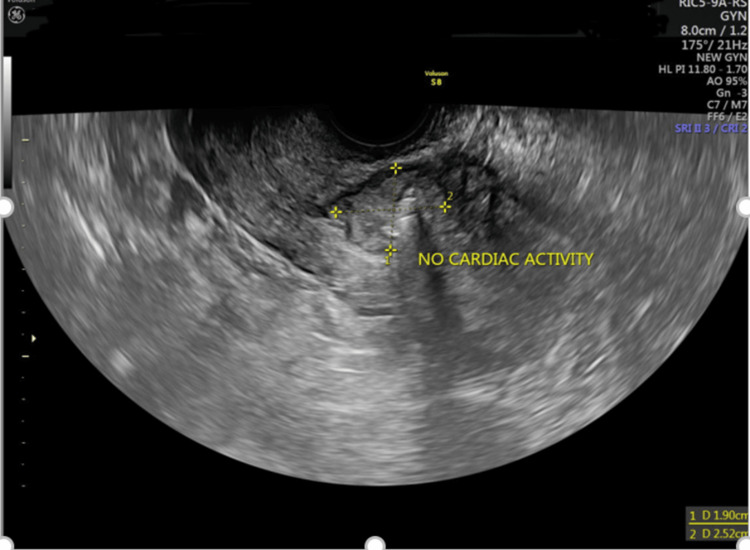
No fetal cardiac activity.

**Figure 3:**
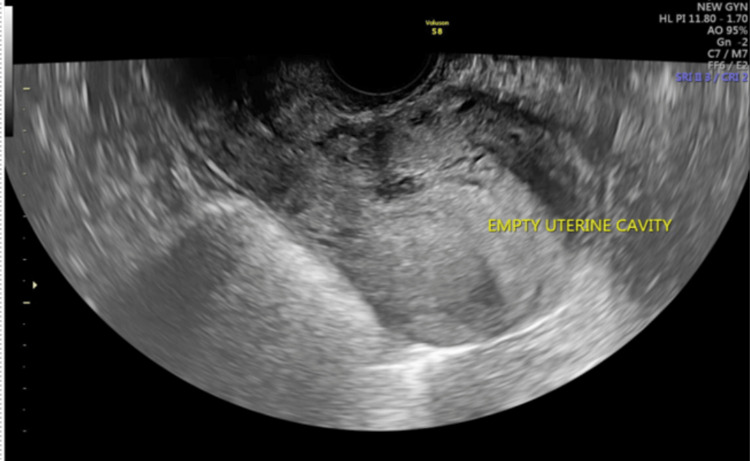
Empty uterine cavity post-procedure.

Beta HCG value of 24,157 IU/L. Informed consent was obtained, and the patient was planned for a multidose methotrexate regimen after assessing her suitability for the regimen. After two doses of methotrexate (given intramuscularly), her beta HCG level was 34,953 IU/L, and a repeat TVS scan showed scar pregnancy with persistent fetal cardiac activity. The patient was planned for intra-sac methotrexate and injection of potassium chloride (KCl). After obtaining informed consent, under TVS guidance, the ectopic scar pregnancy reduction was done by injecting 1 mL of KCl intracardiac administration and 50 mg injection of methotrexate in the gestational sac. No cardiac activity was noted at the end of the procedure (Figure 2). Figure 3 shows an empty uterine cavity post-procedure.

Post-procedure vitals were stable, and a repeat beta HCG after two weeks was found to be <10 IU/L.

## Discussion

Ectopic pregnancy is one of the major causes of maternal morbidity and refers to the implantation of the blastocyst occurring outside of the uterine cavity. Ectopic pregnancy constitutes 1.2-1.4% of all reported pregnancies [1], with the most common site being the ampulla of the fallopian tube (70% of all ectopic pregnancies) [3]. Some of the rare sites of ectopic pregnancy are the ovaries, the abdomen, the cervix, and the scar of a previous cesarean section.

While the incidence of a cesarean scar pregnancy is poorly identified in the literature, the only risk factor that predisposes women to develop a scar ectopic is a history of one or more cesarean deliveries [4]. Ectopic pregnancy is one of the causes of first-trimester bleeding and is also an obstetric emergency due to the risk of rupture. Symptoms of ectopic pregnancy include vaginal bleeding, abdominal cramps, pelvic pain, and other symptoms based on the site of implantation such as bowel and bladder disturbances. A cesarean scar pregnancy, however, has non-specific symptoms making its detection significantly challenging. The rates of cesarean deliveries have more than doubled in India, from 8% of deliveries in 2005 to 17% of deliveries in 2016 [2]. With a rising preference for deliveries via cesarean section among patients and healthcare providers, the incidence of scar ectopic is expected to increase further.

Cesarean scar pregnancy occurs when a blastocyst implants in a microscopic or macroscopic tract on the uterine scar or the “niche” (or dehiscence) left behind by an incision site of the previous cesarean delivery [5]. As the symptoms of cesarean scar pregnancy are relatively vague, prompt diagnosis is crucial to prevent misdiagnosing it as a threatened abortion. The first line of investigation that can be done is a TVS; however, an MRI can also be done. Criteria for diagnosis include a positive pregnancy test, an empty uterine cavity, and a gestational sac with or without a fetal heartbeat. Once the diagnosis is confirmed, there are usually five modalities of treatment that can be undertaken which include expectant management, medical therapy, surgical intervention, uterine artery embolization, or a combination approach [6].

In our case, as the patient was hemodynamically stable and had no significant comorbidities, we proceeded with a conservative approach to treatment by the administration of methotrexate therapy. The two most common regimens in use are intramuscular methotrexate injection either as a multiple-dose regimen of 1 mg/kg of methotrexate every other day, alternating with 0.1 mg/kg of leucovorin rescue, for up to four doses, or a single-dose regimen based on the body surface area (50 mg/m^2^) without leucovorin rescue [7]. Serial beta HCG monitoring is recommended to ensure the successful termination of the pregnancy. In our patient’s case, as the beta HCG remained elevated after methotrexate injections, we pursued the termination of cesarean scar pregnancy using intracardiac KCl injection and intragestational sac methotrexate injection under TVS guidance. In most cases, a single high-dose (100 mg) injection of methotrexate is shown to be safe and effective, while in a few resistant cases with persistent fetal heart rate, intracardiac injection of KCl has proven to be completely curative [7].

## Conclusions

A conservative approach to the management of cesarean scar pregnancy with the adjunct use of KCl can be used to replace surgical intervention in select cases. This approach can reduce morbidity associated with the surgical termination of ectopic pregnancy, especially in young females, prone to recurrent ectopic pregnancies. The adverse effects caused due to systemic dosing of methotrexate can also be alleviated by this mode of treatment. A thorough evaluation of a hemodynamically stable patient including complete blood count, liver function test, and renal function test is advised before the onset of this line of treatment.

## References

[1] Rana P, Kazmi I, Singh R (2013). Ectopic pregnancy: a review. Arch Gynecol Obstet.

[2] Marion LL, Meeks GR (2012). Ectopic pregnancy: history, incidence, epidemiology, and risk factors. Clin Obstet Gynecol.

[3] Timor-Tritsch IE, Monteagudo A, Calì G, D'Antonio F, Kaelin Agten A (2019). Cesarean scar pregnancy: diagnosis and pathogenesis. Obstet Gynecol Clin North Am.

[4] Bhatia M, Banerjee K, Dixit P, Dwivedi LK (2020). Assessment of variation in cesarean delivery rates between public and private health facilities in India from 2005 to 2016. JAMA Netw Open.

[5] Glenn TL, Bembry J, Findley AD, Yaklic JL, Bhagavath B, Gagneux P, Lindheim SR (2018). Cesarean scar ectopic pregnancy: current management strategies. Obstet Gynecol Surv.

[6] Stika CS (2012). Methotrexate: the pharmacology behind medical treatment for ectopic pregnancy. Clin Obstet Gynecol.

[7] Tzafettas JM, Stephanatos A, Loufopoulos A, Anapliotis S, Mamopoulos M, Kalogeropoulos A (1999). Single high dose of local methotrexate for the management of relatively advanced ectopic pregnancies. Fertil Steril.

